# Optimizing microvascular decompression for trigeminal Neuralgia: Addressing vertebrobasilar ectasia challenges – A technical note

**DOI:** 10.1016/j.bas.2025.104264

**Published:** 2025-04-24

**Authors:** Pavlina Lenga, Carola Wieckhusen, Mohammad Mehdi Hajiabadi, Andreas Unterberg, Sandro M. Krieg, Rezvan Ahmadi

**Affiliations:** a Department of Neurosurgery, Heidelberg University Hospital, Heidelberg, Germany; b Medical Faculty of Heidelberg, Heidelberg University Hospital, Heidelberg, Germany; c Division of Operative Pain Management, Department of Neurosurgery, Heidelberg University Hospital, Heidelberg, Germany

**Keywords:** Trigeminal neuralgia, Vertebrobasilar ectasia, Neurovascular compression, Microvascular decompression

## Abstract

**Introduction:**

Trigeminal neuralgia (TN) can be due vertebrobasilar ectasia (VBE), where elongated, tortuous arteries compress the trigeminal nerve, making surgical management challenging.

**Research question:**

Does a refined microvascular decompression (MVD) technique using a “Teflon cloud” interposition offer sustained symptom relief and medication discontinuation in VBE-induced TN?

**Materials and methods:**

Three patients with VBE-induced TN were treated between 2017 and 2024. Diagnosis was confirmed by MRI/MRA. MVD with a “Teflon cloud” was performed to cushion the nerve without extensive arterial manipulation. Postoperative outcomes were tracked over an 8-month follow-up.

**Results:**

All patients showed immediate, complete relief of TN symptoms, with no recurrences. They discontinued TN-related medications within three months, and no significant complications occurred.

**Discussion and conclusion:**

These findings suggest that interposition-based MVD using a Teflon cloud effectively addresses TN in the context of VBE by providing stable nerve decompression and minimizing vascular manipulation. A refined MVD with a “Teflon cloud” interposition provides safe, sustained relief for VBE-induced TN, warranting further investigation in larger patient cohorts.

## Highlights

• This retrospective analysis demonstrates a refined microvascular decompression approach, effective for vertebrobasilar ectasia-induced trigeminal neuralgia.
• All three patients achieved immediate, complete relief of symptoms, with no recurrence noted over an eight-month follow-up period.
• No significant postoperative complications occurred, and all patients successfully discontinued their trigeminal neuralgia medications within three months.

## 1 Introduction

Trigeminal neuralgia (TN) is a common neurological disorder characterized by sudden, severe episodes of unilateral facial pain, often triggered by benign stimuli (Cruccu et al., 2016; Trigeminal neuralgia – diagnosis and treatment - Stine Maarbjerg et al., 2017). The prevailing hypothesis for classical TN posits that it arises from nerve compression by vascular structures, most commonly the superior cerebellar artery (SCA) and the anterior inferior cerebellar artery (AICA), along with their branches. Occasionally, venous compressions are also implicated. A less frequent cause is vertebrobasilar dolichoectasia (VBE), also known as vertebrobasilar ectasia (VBE), a distinct condition marked by elongated and tortuous vertebrobasilar arteries, which exert pressure on the trigeminal nerve (Smoker et al., 1986; Sun et al., 2017a; Yuan et al., 2014). Although rare, with VBE-related TN accounting for only 2–7 % of all cases, its impact is significant (Honey and Kaufmann, 2018; Linskey et al., 1994; Sun et al., 2017b; Yang et al., 2012).

Traditional surgical treatments—including rhizotomy, coagulation, and nerve repositioning—have been employed to alleviate symptoms; however, their efficacy is often limited and remains a subject of debate (Ahmadi and Tronnier, 2024) (7–9,11). In particular, cases involving vertebrobasilar elongation (VBE) present unique challenges due to the elongation and tortuosity of megadolichobasilar vessels. This elongation leads to compression of the trigeminal nerve that is difficult to alleviate surgically. The pathophysiology involves atherosclerotic changes that make the vessels fragile and less amenable to repositioning during microvascular decompression (MVD). Additionally, the proximity of these elongated vessels to other cranial nerves increases the risk of complications. Consequently, the efficacy of MVD in TN cases associated with VBE remains underexplored, and surgical intervention requires careful consideration of these anatomical and pathological complexities.

In this context, we document our experiences with three patients at one of Germany's leading national centers for the treatment of TN due to VBE, underscoring the safety and efficacy of MVD. This study details our refined surgical technique, which emphasizes meticulous dissection and mobilization to safely reposition the compressive artery away from the trigeminal nerve, thereby alleviating symptoms and potentially offering a durable solution for patients suffering from this debilitating condition.

## 2 Methods

### 2.1 Study design and participants

This retrospective study analyzed a cohort of three patients treated at our institution between September 2017 and November 2024. This study was approved by the local ethics committee of our institution (S 383/2024) and conducted in accordance with the Declaration of Helsinki. The necessity for informed consent was dismissed due to the study's retrospective design.

**Fig. 1:**
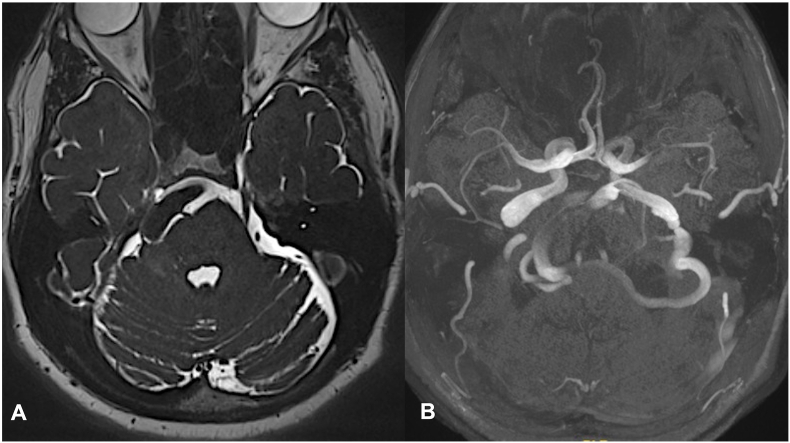
A. Axial T2-weighted magnetic resonance image demonstrating a prominent basilar artery ventral to the brainstem. The artery appears to be compressing the anterolateral aspect of the right side of the pons, near the expected location of the root entry zone of the right trigeminal nerve. B. 3D time-of-flight MR angiography showing a dilated and tortuous vertebrobasilar artery system. The vessel is deviating and curving laterally toward the presumed root entry zone of the right trigeminal and facial nerves, which may contribute to neurovascular compression.

Participants consisted of all consecutive cases presenting to our institution and were included based on the following inclusion criteria: diagnosis of trigeminal neuralgia (TN) attributed to vertebrobasilar ectasia (VBE) as confirmed by MRI and MR angiography, as shown in Fig. 1, and failure to respond adequately to pharmacological treatments.

Diagnostic Imaging MRI and MR angiography were performed to confirm the diagnosis of TN and to visualize the anatomical details of the vertebrobasilar ectasia impacting the trigeminal nerve. Imaging studies focused on identifying the location and extent of vascular compression.

### 2.2 Surgical procedure

All surgical interventions were performed by a team of experienced neurosurgeons (RA, SK, AU) using the microvascular decompression (MVD) technique. Patients were positioned in the park-bench position under general anesthesia. A right or left retrosigmoidal craniotomy was performed, followed by dural opening to access the cerebellopontine angle. Cerebrospinal fluid was aspirated from the cisterna magna to improve operative field visibility. The offending arterial and venous structures compressing the trigeminal nerve were identified, and meticulous dissection was conducted. Decompression was achieved using Teflon/Gore Tex “cloud” interposed between the nerve and compressive vessels without mobilizing the artery significantly.

### 2.3 Postoperative care and follow-up

The primary outcome measured was the resolution of TN symptoms, with follow-up appointments scheduled at a median of 3 months post-surgery to assess pain levels and any neurological deficits. The effectiveness of the surgery was assessed based on the reduction or elimination of TN symptoms and the discontinuation of TN medication.

### 2.4 Statistical analysis

Descriptive statistics were used to summarize patient demographics, surgical outcomes, and follow-up data. Given the small sample size, inferential statistics were not applicable.

## 3 Results

### 3.1 Case 1

A 75-year-old male patient presented to our outpatient clinic with a three-year history of trigeminal neuralgia, experiencing severe pain in the V2 and V3 territories of the trigeminal nerve. The pain, described as sharp and electrical, was exacerbated by activities such as talking and chewing, and occasionally occurred spontaneously. Despite high doses of carbamazepine and amitriptyline, his symptoms were inadequately controlled. The patient's medical history included arterial hypertension, which was being managed with three different antihypertensive medications. Diagnostic imaging with MRI and MR angiography revealed mechanical compression of the right trigeminal nerve due to vertebrobasilar dolichoectasia, leading to the decision to perform surgery. Intraoperatively, severe pulsating compression from an enlarged, tortuous vertebral artery and multiple thick dorsal veins was noted. Strategic placement of Teflon sponges around the nerve provided thorough decompression. Postoperatively, the patient experienced immediate resolution of his lancinating facial pain. At the eight-month follow-up, he remained pain-free and had successfully discontinued all medications for trigeminal neuralgia, including carbamazepine and amitriptyline.

### 3.2 Case 2

A 57-year-old patient presented with typical TN symptoms affecting the V1 and V2 territories of the fifth cranial nerve on the right side, with a symptom duration of two years. The patient described the pain as being triggered by touch, chewing, and speaking, with initial onset noted upon touching the right nose/upper lip area. Over time, the symptoms progressively worsened. Since March 2024, the patient had been **taking carbamazepine**, which initially alleviated the symptoms; however, over time, increasingly higher doses were required, leading to significant side effects. No comorbidities were known, although the patient experienced trochlear nerve dysfunction, presenting as diplopia. MRI revealed an elongated and right-sided basilar artery, likely compressing the cranial nerves in this region. Involvement of the trochlear and vestibulocochlear nerves was also considered probable. Given these findings, surgical intervention was indicated. During surgery, markedly enlarged and arteriosclerotic vertebrobasilar arteries were seen compressing the trigeminal nerve and adjacent cranial nerves. Careful neurolysis allowed effective decompression using Teflon interposition. Postoperatively, the patient experienced dizziness and nausea, but postoperative CT imaging showed no pathological findings. By the time of discharge, the patient's symptoms had completely resolved. Three months following the surgery, the patient remained pain-free, and carbamazepine was successfully discontinued.

### 3.3 Case 3

A 69-year-old male with arterial hypertension presented with a four-month history of acute, left-sided trigeminal neuralgia (TN), characterized by electric and stabbing pain exacerbated by facial touch. Despite pharmacological treatment with paracetamol, dolantin, and phenytoin, symptom control was inadequate. Diagnostic MRI and MR angiography revealed an elongated basilar artery compressing the left trigeminal nerve, indicative of basilar dolichoectasia. The severity of symptoms and the failure of medical management necessitated surgical intervention. A left suboccipital craniotomy was performed with the patient in the park-bench position. Intraoperatively, significant compression by an arteriosclerotic basilar artery was confirmed. Teflon sponges were strategically positioned, decompressing the nerve effectively without extensive manipulation of the artery. The patient reported immediate alleviation of TN symptoms post-surgery. Follow-up assessments over one year showed no recurrence of pain, allowing for the discontinuation of all analgesic medications. The postoperative period was uncomplicated, with the patient experiencing no new neurological deficits or other adverse effects.

**Fig. 2:**
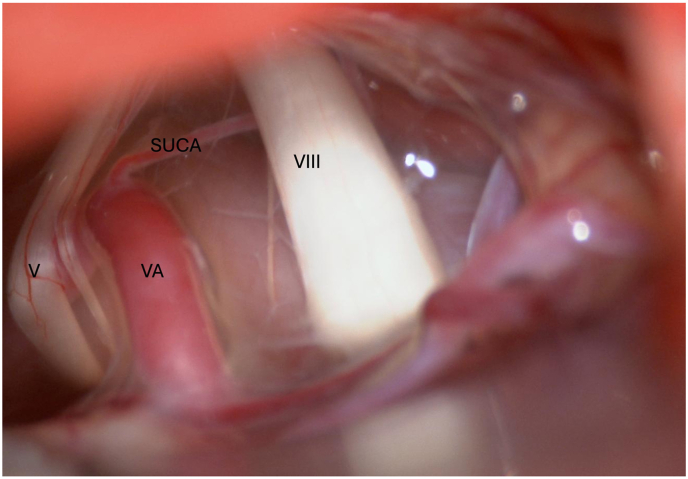
The vertebral artery (VA) is compressing and distorting both the trigeminal (V) and vestibulocochlear (VIII) nerves. The superior inferior cerebellar artery (SUCA) is observed away from both nerves.

Fig. 2 presents an intraoperative image of a the ectatic VA significantly compressing the trigeminal nerve.

## 4 Discussion

In this study, we examined three middle-aged patients diagnosed with TN caused by VBE. Notably, all participants in our cohort had a history of arterial hypertension and had been receiving medication for TN for more than three years. Each patient underwent MVD, which involved the careful opening of the arachnoid membranes around the affected nerves and vessels. This procedure was designed to enhance the flexibility of the area around the fragile and atherosclerotic VBE. We strategically placed Teflon clouds between nerve and artery to ensure effective decompression, resulting in immediate postoperative resolution of TN symptoms without neurological deficits. By the median 8-month follow-up, patients remained symptom-free, underscoring the technique's immediate effectiveness in this complex pathology.

Our results align with broader research indicating the relative rarity of VBE-related TN. For instance, in a cohort study by Wang et al., only 6 out of 110 TN cases were attributed to VBE, making up 5.5 % of their sample. Similarly, Linskey et al. reported a 2.4 % incidence (31 out of 1400 cases) of TN caused by VBE (Linskey et al., 1994). Ma et al. found a prevalence of 2.40 % in a dedicated study of 458 patients with VBE-induced TN (Ma et al., 2013), while Yang et al. reported a prevalence of 2.0 % in a slightly larger sample of 475 cases (Yang et al., 2012). These studies collectively underscore the infrequency of TN resulting from VBE and highlight the ongoing challenges in its surgical management. Significantly, across these studies, there was a male predominance in patients aged between 47 and 75 years, with a notable duration from diagnosis to treatment ranging from 2.2 to 6.8 years. The male predominance observed aligns with our findings where all three patients were male, averaging 64 years in age, and similarly suffering from arterial hypertension. The vertebral artery was identified as the primary offending vessel in about 34.5 % of cases across the literature, with the basilar artery implicated in approximately 64.1 % of cases and the vertebrobasilar junction in about 1.41 %. This study contributes valuable insights into the surgical management of TN caused by VBE, particularly in patients with concurrent arterial hypertension. The 10-month follow-up without recurrence underlines MVD's potential as a durable therapeutic option for this complex clinical scenario. Given the limited patient numbers and complexities inherent to this condition, our detailed description of a reproducible surgical approach contributes substantially toward managing these rare cases.

Managing trigeminal neuralgia (TN) caused by vertebrobasilar ectasia (VBE) through microvascular decompression (MVD) poses significant surgical challenges due to the complex vascular anatomy involved. The ectatic and tortuous nature of the vertebrobasilar arteries in VBE complicates surgical access and manipulation, as these vessels are often fragile and atherosclerotic, increasing the risk of intraoperative vascular injury (Pico et al., 2015; Yu et al., 1982). Several surgical techniques have been proposed to address these challenges, including the transposition of the offending vessel. El-Ghandour (2010) emphasized the importance of displacing the vessel sufficiently lateral to the Obersteiner-Redlich zone—approximately 2.2 mm from the trigeminal root entry zone—to achieve reliable pain relief and prevent symptom recurrence. Similarly, Yang et al. (2012) reported good outcomes, achieving an immediate 80 % success rate by moving the vertebrobasilar artery (VBA) proximally using a lateroinferior cerebellar approach during MVD. However, many surgeons remain skeptical about the feasibility of transposition techniques in VBE cases. Mobilizing the large and tortuous VBA is inherently difficult due to its significant tortuosity, hypertrophy, and sclerosis (Linskey et al., 1994). Such extensive neurovascular manipulation increases the risk of intraoperative complications, including vasospasm of small arteries leading to brainstem ischemia and potential infarction resulting from damage to perforating vessels or alterations in blood flow that may promote thrombus formation in the ectatic, atherosclerotic VBA. Additionally, repositioning the VBA can inadvertently cause new postoperative neurological deficits by mechanically compressing adjacent cranial nerves (fourth, sixth, or eighth nerves) along its new anatomical course. Given these limitations, we advocate for an alternative surgical approach tailored to the unique challenges of VBE-associated TN. In our experience, thoroughly opening the arachnoid membranes surrounding the nerves and vessels—from the lower cranial nerves up to the trigeminal nerve—is crucial. This step enhances the mobility of the VBA by relieving the restrictions imposed by the arachnoid, rather than attempting to overcome the vessel's inherent rigidity. If the VBA cannot be easily mobilized, we recommend gently inserting Teflon felt between the nerve and artery to gradually separate them, thereby achieving decompression without extensive manipulation of the vessel. To ensure that no contributing vessels are overlooked, it is essential to explore the entire length of the trigeminal nerve, including the cisternal segment and the root entry zone (REZ), to identify any small offending vessels such as the superior cerebellar artery (SCA) or anterior inferior cerebellar artery (AICA). In our practice, we have found that an additional selective partial posterior rhizotomy is unnecessary if effective isolation between the nerve and vessel is achieved intraoperatively, as previously described (Sun et al., 2017b).

Microvascular decompression (MVD) for trigeminal neuralgia (TN) caused by vertebrobasilar ectasia (VBE) introduces significant surgical complexities, especially concerning the manipulation of the ectatic and tortuous vertebrobasilar arteries (VBA). These arteries often exhibit pronounced atherosclerosis and fragility, heightening the risk of intraoperative vascular damage (Pico et al., 2015; Yu et al., 1982). El-Ghandour et al. have emphasized the need for the vessel to be displaced laterally beyond the Obersteiner-Redlich zone—approximately 2.2 mm from the trigeminal root entry zone—to secure both effective pain relief and recurrence prevention (El-Ghandour, 2010). Yang et al. also reported successful outcomes with proximal mobilization of the VBA using a lateroinferior cerebellar approach, achieving an 80 % immediate relief rate after MVD (Yang et al., 2012). Despite these successes, the inherent characteristics of the VBA in VBE cases—such as extreme tortuosity and sclerosis—make such maneuvers exceedingly challenging. Linskey et al. highlighted the considerable difficulty in mobilizing the VBA, which involves extensive neurovascular manipulation, potentially leading to severe complications such as vasospasm of small arteries and subsequent brainstem ischemia or even infarction (Linskey et al., 1994). The risk of new postoperative neurological deficits due to mechanical compression of neighboring cranial nerves (such as the fourth, sixth, or eighth) in their altered anatomical pathways is also a serious concern. Our clinical experience underscores the critical nature of fully opening the arachnoid membranes around the nerves and vessels to improve the VBA's mobility. When direct mobilization proves problematic, we have found that carefully positioning Teflon wadding into the spaces between the nerve and artery can effectively segregate them without necessitating aggressive repositioning of the artery. Additionally, thorough exploration of the entire length of the trigeminal nerve, including the cisternal segment and the root entry zone (REZ), ensures that no potentially compressive vessels are overlooked, such as the superior cerebellar artery (SCA) and anterior inferior cerebellar artery (AICA). Moreover, we deem an additive selective partial posterior rhizotomy unnecessary if satisfactory isolation between the nerve and vessel is achieved intraoperatively (Sun et al., 2017b). Although arterial transposition has shown good outcomes in smaller, more malleable vessels, Uhl et al.‘s single, inadequately described case involving a large ectatic artery underscores potential safety and feasibility concerns, reinforcing the appropriateness of our interposition technique. (Uhl et al., 2024). While effective for smaller and more malleable arteries, its application in cases of VBE-associated TN is fraught with increased complexity and risks. Indeed, in Uhl et al.‘s study, the singular case of VBE-associated TN was not detailed, suggesting potential feasibility and safety concerns with the transposition method in such instances. This lack of detailed reporting underscores the necessity for exploring alternative surgical strategies. Given the anatomical and pathological challenges posed by the ectatic VBA, the risks associated with extensive vessel manipulation—ranging from vascular injury and brainstem ischemia to thrombus formation and postoperative neurological impairments—make the transposition technique less desirable. Instead, the interposition technique stands out as a safer and more effective alternative, providing successful decompression without the complications associated with extensive vessel mobilization. This method minimizes the manipulation of the VBA, thereby reducing the likelihood of intraoperative and postoperative complications, making it a preferable option in managing TN associated with VBE.

### 4.1 Limitations

Our study has several key limitations, including a small sample size that limits generalizability, a short follow-up period, and a homogeneous patient demographic of middle-aged males with arterial hypertension. These factors may not adequately represent the broader population affected by TN due to VBE. Additionally, the lack of a control group prevents a definitive comparison of surgical techniques. Despite these limitations, our findings provide a technical foundation for future prospective studies, suggesting that the interposition technique could be a safer and potentially more effective surgical option for managing TN associated with VBE. Future research should aim to include larger and more diverse populations, as well as longer follow-up periods to validate and refine the efficacy of this approach.

## 5 Conclusions

Although manipulating an ectatic vertebrobasilar artery complex poses significant surgical challenges, our study indicates that MVD remains an effective therapy for TN when a proper surgical strategy is implemented. By employing meticulous techniques—such as thoroughly opening the arachnoid membranes to enhance vessel mobility and utilizing the interposition method to gently separate the artery from the nerve without extensive manipulation—a successful surgical outcome is achievable. This approach minimizes the risks associated with vessel mobilization, particularly in cases involving VBE. Our findings support the efficacy of MVD in providing symptom relief for patients with TN caused by VBE. Future studies with larger patient populations are necessary to confirm these results and to further optimize surgical strategies for this complex condition.

## Consent to participate

Due to retrospective nature of the study an informed consent was waived.

## Ethics approval

This study was conducted in accordance with the Declaration of Helsinki and approved by the local ethics committee (S 383/2024).

## Consent for publication

No individual person's data were included in this study.

## Data material availability

The datasets generated during and/or analyzed during the current study are available from the corresponding author on reasonable request.

## Human and animal ethics

Not applicable.

## Funding

There was no external funding for the presented work.

## Declaration of competing interest

The authors have no relevant financial or non-financial interests to disclose.
