# Supplementary material for: Optimizing microvascular decompression for trigeminal Neuralgia: Addressing vertebrobasilar ectasia challenges – A technical note
Source: Brain Spine. 2025 Apr 24;5:104264. doi: 10.1016/j.bas.2025.104264 (PMC12127552; doi:10.1016/j.bas.2025.104264)
Supplement: Multimedia component 1 [file mmc1.docx]

| **Variable** | **Case 1** | **Case 2** | **Case 3** |
| --- | --- | --- | --- |
| **Age (years)** | 75 | 57 | 69 |
| **Gender** | Male | Male | Male |
| **Symptom duration** | 3 years | 2 years | 4 months |
| **Affected TN territories** | V2, V3 | V1, V2 | Left-sided TN |
| **Trigger factors** | Talking, chewing, spontaneous | Touch, chewing, speaking | Facial touch |
| **Comorbidities** | Arterial hypertension | Trochlear nerve dysfunction (Diplopia) | Arterial hypertension |
| **Preoperative medication** | Carbamazepine, Amitriptyline | Carbamazepine | Paracetamol, Dolantin, Phenytoin |
| **Radiological findings** | Compression by enlarged, tortuous vertebral artery | Elongated arteriosclerotic basilar artery compressing nerves V–X | Basilar dolichoectasia compressing left trigeminal nerve |
| **Intraoperative findings** | Severe compression (vertebral artery and dorsal veins) | Marked compression by vertebrobasilar artery | Significant compression by arteriosclerotic basilar artery |
| **Surgical Technique** | Teflon sponge interposition | Teflon interposition | Teflon sponge interposition |
| **Immediate postoperative outcome** | Complete resolution of pain | Initial dizziness/nausea, rapid resolution of symptoms | Immediate symptom resolution |
| **Follow-up duration** | 8 months | 3 months | 12 months |
| **Long-term outcome** | Pain-free, medications discontinued | Pain-free, medications discontinued | Pain-free, medications discontinued |

**Supplement Table 1:** Comprehensive Clinical, Radiological, and Operative Findings of Three Patients with Vertebrobasilar Ectasia-Induced Trigeminal Neuralgia Treated by Microvascular Decompression
